# Combination therapy of RY10-4 with the γ-secretase inhibitor DAPT shows promise in treating HER2-amplified breast cancer

**DOI:** 10.18632/oncotarget.6769

**Published:** 2015-12-26

**Authors:** Feng Su, Shilin Zhu, Jinlan Ruan, Yagmur Muftuoglu, Longbo Zhang, Qianying Yuan

**Affiliations:** ^1^ Department of Emergency, Xiangya Hospital, Central South University, Changsha, China; ^2^ The Second Affiliated Hospital, Hunan University of Chinese Medicine, Changsha, China; ^3^ Department of Pharmacology, Huazhong University of Science and Technology, Wuhan, China; ^4^ Department of Pharmacology, Yale University School of Medicine, New Haven, CT, USA; ^5^ Department of Neurosurgery, Xiangya Hospital, Central South University, Changsha, China

**Keywords:** RY10-4, resistance, Notch, breast cancer

## Abstract

RY10-4, a novel protoapigenone analog, shows potent cytotoxicity against human breast cancer cells. However, breast cancer cell lines overexpressing human epidermal growth factor receptor 2 (HER2), SKBR3 and BT474, showed less sensitivity to RY10-4 when compared to breast cancer cells lines expressing lower levels of HER2, such as MDA-MB-231 and MCF-7 cells. This was associated with aberrant hyperactivity in Notch signaling in cells treated with RY10-4, since treatment with RY10-4 causes an increase in Notch activity by 2-to3.5-fold in SKBR3 and BT474 cell lines. The increase in activity was abrogated with a γ-secretase inhibitor, DAPT, or with Notch1 small-interfering RNA (si-Notch1). Cell proliferation was inhibited more effectively by RY10-4 plus DAPT or si-Notch1 than either agent alone. RY10-4 plus DAPT increases apoptosis in both HER2-overexpressing cell lines by two-fold compared to RY10-4 alone, while DAPT alone has no significant effects on apoptosis. In addition, we previously found RY10-4 could inhibit tumor growth through the PI3K/AKT pathway. Here we report that the combination of RY10-4 and DAPT exhibit additive suppression on AKT phosphorylation, contributing to the anti-cancer effects. In an animal model, this combination therapy inhibits the growth of SKBR3 tumor xenografts in nude mice to a greater extent than treatment with either reagent alone. These results indicate that the aberrant activation of Notch signaling impedes the inhibitory effect of RY10-4 on HER2-amplified cell proliferation. Furthermore, these adverse effects can be prevented by treatment combining RY10-4 with a Notch pathway inhibitor.

## INTRODUCTION

Breast cancer is the most commonly diagnosed malignancy among women, and it is a leading cause of cancer death in females from western countries [1]. Novel strategies for prevention and treatment of breast cancer are needed to minimize off-target drug-related toxicities and to ultimately enhance patient outcomes. Protoapigenone (Figure S1A), isolated from torres's ferns [2, 3] is a flavonoid that displays potent antitumor activity against a broad spectrum of human cancer cell lines [2, 4, 5]. This compound contains an unusual nonaromatic B-ring. Based on this, we developed a novel compound RY10-4 (Figure S1B, Publication Number: CN 102731456 B), which is structurally related to protoapigenone but which exhibits better antitumor activity and fewer side effects compared to protoapigenone *in vitro* and *in vivo* [6]. Our recent studies show that different human breast cancer cell lines display variable sensitivity to RY10-4. RY10-4 exhibits comparable growth-inhibitory effects on the triple-negative cell line MDA-MB-231 and the estrogen receptor (ER)-positive cell line MCF-7. The HER2-positive cell lines SKBR3 and BT474 exhibit similar inhibitory effects but less sensitivity than the other two.

Notch signaling is one of the most important signaling cascades involved in drug resistance in tumor cells. Notch genes encode transmembrane receptors that are highly conserved from invertebrates to mammals. These receptors interact with ligands expressed by adjacent cells to regulate cell fate specification, differentiation, proliferation, and survival [7]. The Notch system in vertebrates is comprised of four receptors (Notch1-4) and at least five ligands from the families Delta and JAG/Serrate (DSL): Delta-like(Dll)-1, Dll-3, Dll-4, JAG1, and JAG2 [8, 9]. In breast cancer patients who received tomoxifen treatment, the activity of Notch signaling in tumor tissue correlates with drug resistance and poor prognosis [10]. Also, in a mouse model, the Notch1 pathway promotes acquired resistance to tamoxifen in serially passaged breast cancer xenografts [11]. Similar drug resistance to Adriamycin, Cisplatin, Etoposide, and Taxol were reported in breast cancer cells and lymphoblastic leukemia cells, both due to intracellular Notch1 signaling [12]. Additionally, treating mice with a Notch inhibitor restores tamoxifen sensitivity, and inhibiting glucocorticoid-resistant T-cell acute lymphoblastic leukemia cell lines sensitized to Notch-1 lead to glucocorticoid-induced apoptosis [10, 13]. Most interestingly, other groups found that inhibition of Notch signaling results in downregulation of HER2 expression, while the expression of activated Notch1 and Hes1 is significantly increased after treatment with trastuzumab, a HER2 inhibitor [14, 15]. This indicates that Notch signaling occurs upstream of HER2 signaling, and HER2 negatively regulates Notch expression.

Based on our previous data reporting that RY10-4 inhibits HER2 expression in SKBR3 cells, we propose that decreased HER2 expression induces hyperactive Notch signaling, a possible mechanism of drug resistance caused by RY10-4 treatment. Here, we report aberrant hyperactive Notch signaling in HER2-overexpressing cells SKBR3 and BT474 in response to RY10-4 treatment, opposing the apoptotic effects of RY10-4. Inhibition of Notch signaling by the γ-secretase blocker DAPT or siNotch1 sensitizes breast cancer cells to RY10-4 *in vitro* and *in vivo*. Thus, our data illustrates that RY10-4 holds promising anti-tumor activity against triple-negative breast cancer and ER-positive breast cancer. In combination with a Notch inhibitor, RY10-4 offers a new opportunity in HER2-positive breast cancer therapy.

## RESULTS

### HER2-negtive breast cancer cells are more sensitive to RY10-4

**Figure 1:**
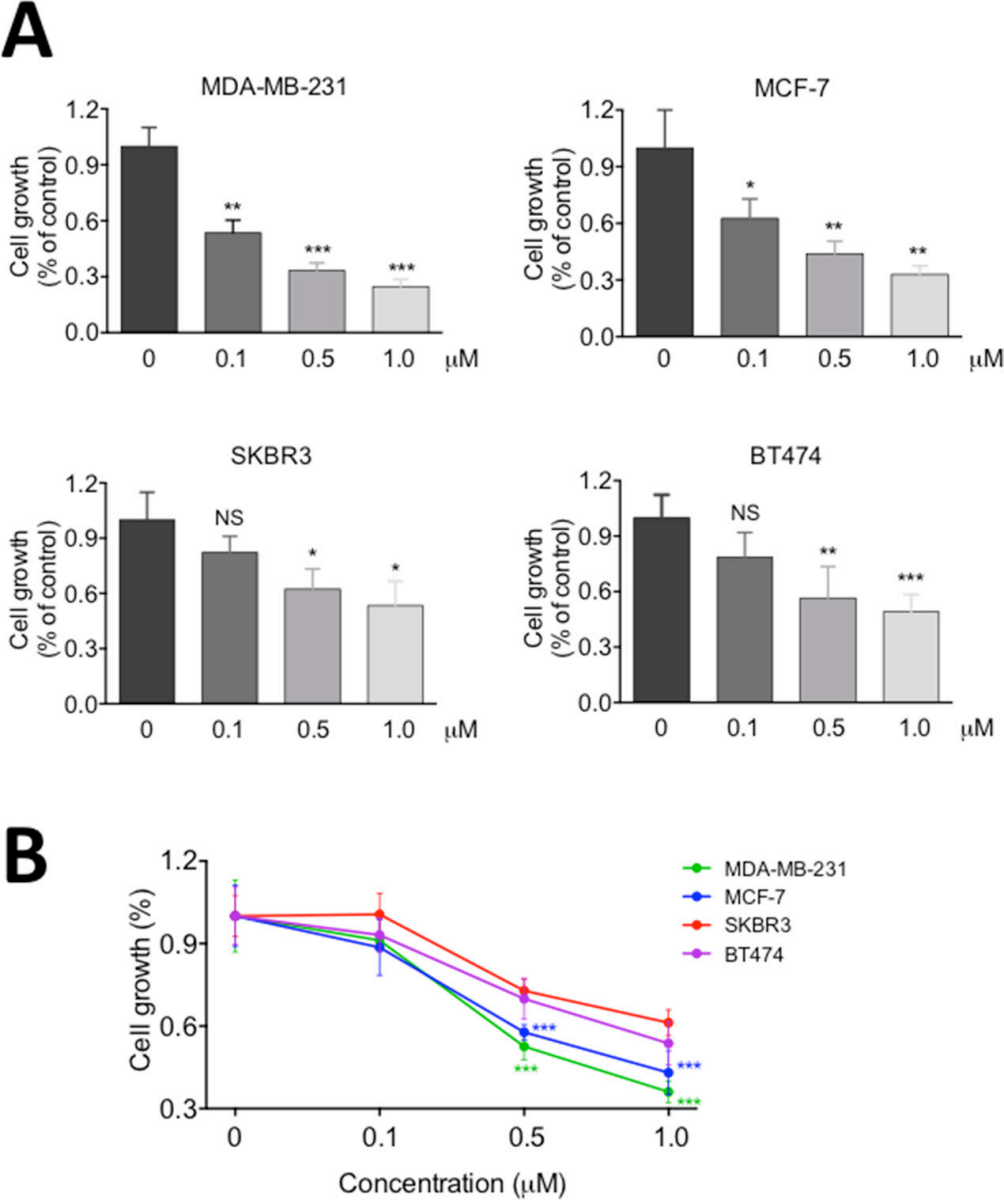
RY10-4 inhibits the proliferation of breast cancer cells Four breast cancer cell lines (MDA-MB-231, MCF-7, SKBR3, BT474) were treated with 0.1, 0.5, and 1.0 μM RY10-4 for 24 h. (**A**) Monolayer growth rates of cells were determined by the methylene blue assay. (**B**) Monolayer growth rates of cells were determined by the MTT assay. Data represent mean ± SD, *P < 0.05, **P < 0.01, ***P < 0.001, versus vehicle control.

In the previous studies [6, 16], we have shown that RY10-4 is a potent anti-tumor compound against breast cancer cells, but the HER-2 overexpressing SKBR3 cell line proves less sensitive to RY10-4 than cell lines expressing lower levels of HER2, such as MDA-MB-231 and MCF-7 cells. To specifically address this question, we treated two commonly used HER2-overexpressing breast cancer cell lines, SKBR3 and BT-474, with RY10-4 for 24 hours. We further evaluated the anti-proliferation effects of RY10-4 using two independent colorimetric assays, methylene blue (Figure 1A) and tetrazolium salt (MTT, Figure 1B). Both HER2-negtive cell lines MDA-MB-231 and MCF-7 were more sensitive to RY10-4. Cell proliferation in this cell lines was inhibited about two-fold compared to the SKBR3 and BT-474 cell lines.

### RY10-4 increases Notch-1 transcriptional activity and expression of endogenous Notch targets in HER2-amplified breast cancer cells

**Figure 2:**
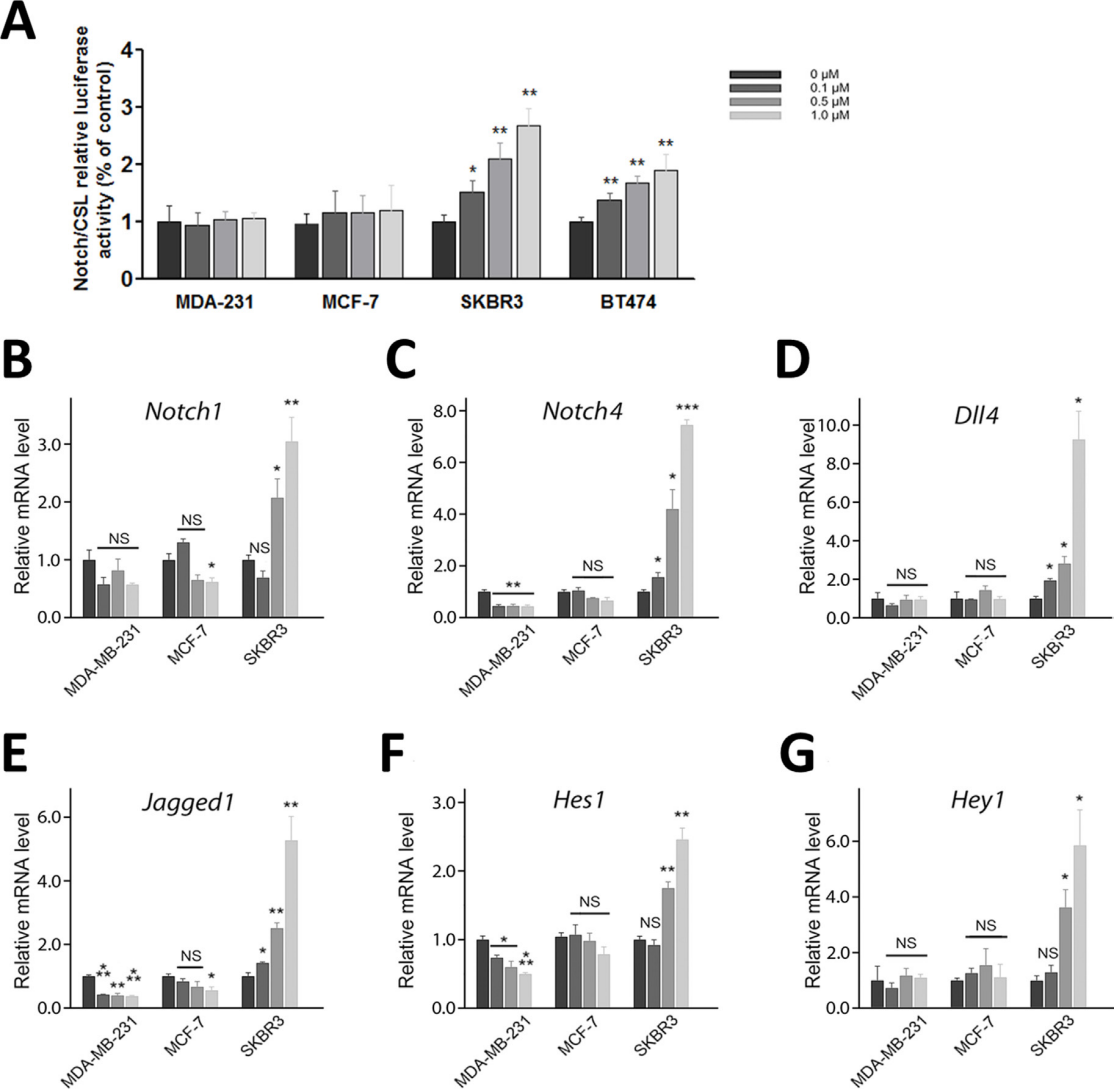
Treatment of HER2-amplified breast cancer cells with RY10-4 induces Notch signaling (**A**) Measurement of CSL luciferase reporter activity in MDA-MB-231, MCF-7, SKBR3, and BT474 cells after 6 h of RY10-4 treatment. (**B–G**) qPCR analysis of Notch1, Notch4, Dll4, Jagged1, Hes1, and Hey1 gene expression in MDA-MB-231, MCF-7, and SKBR3 cells in the absence and presence of RY10-4 over a 6-h time course. Expression of Notch signaling genes was normalized to GAPDH levels. The data represent mean ± SD, *P < 0.05, **P < 0.01, ***P < 0.001, versus vehicle control.

Since activation of Notch signaling in response to HER2 targeted treatment is responsible for drug resistance [17, 18], we first examined Notch activity in four breast cancer cells lines (SKBR3, BT474, MCF-7, and MDA-MB-231) in response to RY10-4 treatment. The results show that treatment with RY10-4 increases Notch transcriptional activity three-fold in SKBR3 and two-fold in BT-474 compared to MDA-MB-231 and MCF-7 cells (Figure 2A), as measured by a C protein binding factor 1/Suppressor of Hairless/Lag1 (CSL) reporter assay.

**Figure 3:**
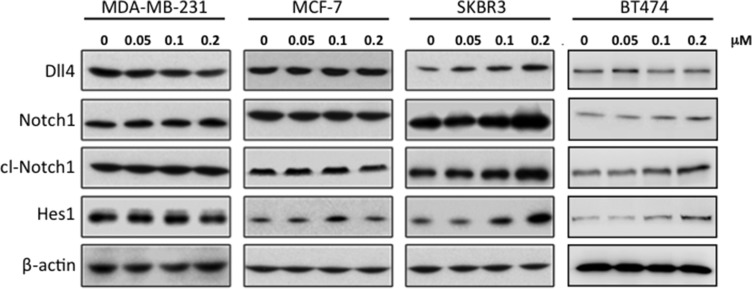
Treatment of SKBR3 cells with RY10-4 leads to increased levels of proteins involved in Notch signaling Western blot analysis of Dll4, Notch1, NICD (cleaved Notch-1 Val-1744 antibody), and Hes1 in MDA-MB-231, MCF-7, SKBR3, and BT474 cells treated with RY10-4 for 24 h. This shows a concentration-dependent increase in Notch protein levels in SKBR3 cells but no significant change in MDA-MB-231 and MCF-7 cells.

We then measured expression of Notch and downstream target mRNAs in these cell lines in response to RY10-4 treatment. Both HER2-overexpressing cell lines, SKBR3 and BT474, show increased expression of Notch receptor genes (Notch1 and Notch4) and Notch target genes (Hes1 and Hey1) after RY10-4 treatment. Although RY10-4 increases Notch signaling in both HER2-positive cell lines, the expression of genes for Notch ligands is slightly reduced or unchanged only in BT474 cells. This discrepancy may be explained by the existence of different molecule footprints of different breast cancer subtypes [19]. Expression of Notch signaling genes is decreased or unchanged in MCF-7 cells and MDA-MB-231 cells (Figure 2B–2G and Figure S2). Furthermore, RY10-4 treatment causes elevated protein expression of the endogenous Notch target Hes1 and activated Notch1 (cleaved Notch1) proteins by two-to-eight-fold in SKBR3 cells and BT474 cells. However, in MDA-MB-231 and MCF-7 cells, protein levels remain unchanged upon RY10-4 treatment (Figure 3). These results suggested that RY10-4 increases Notch activity in HER2-positive breast cancer cells.

In addition, we tested MAPK and Wnt signaling pathways in SKBR3 cells to determine whether RY10-4 affects multiple signaling pathways involved in cell survival. Interestingly, protein levels of phospho-p38, phospho-JNK, phospho-LRP6, and wnt3a do not change in response to RY10-4 treatment (Figure S3). These results indicate that RY10-4 specifically increases activity of the Notch signaling pathway.

### Down-regulation of Notch sensitizes HER-2 positive breast cancer cells to RY10-4

**Figure 4:**
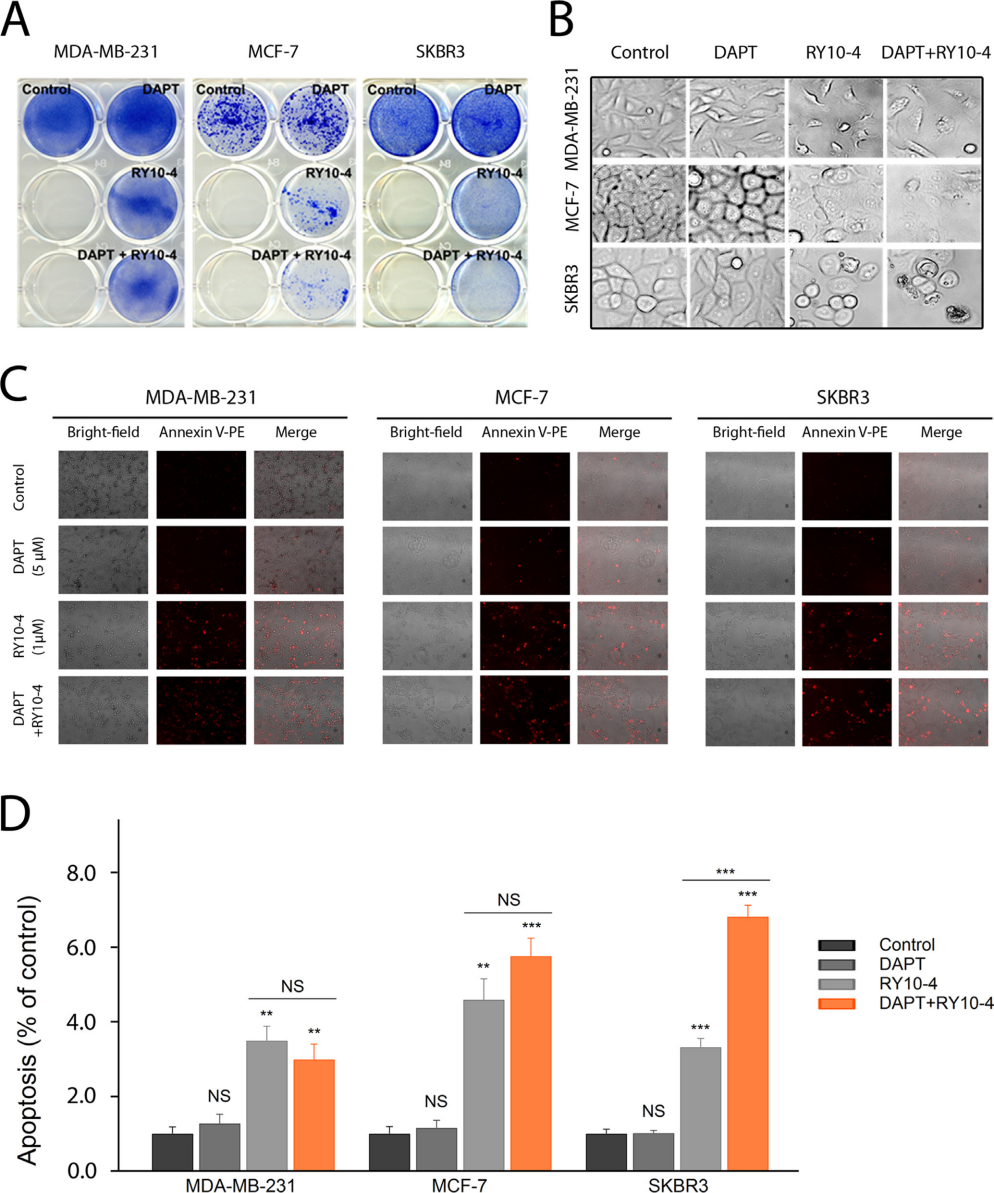
DAPT and RY10-4 show synergy in inhibiting cell proliferation *in vitro* (**A**) The effect of DAPT (5 μM), RY10-4 (1 μM), and combination of the two on the growth of MDA-MB-231, MCF-7 and SKBR3 was investigated using the colony formation assay. The two left wells of each plate were left empty. (**B**) Representative micrographs of the four breast cancer cell lines with 5 μM DAPT treatment, 1 μM RY10-4 treatment, and combinational treatment for 24 h. (**C**) Annexin-PE staining was used to evaluate cell viability following treatment of different samples. Morphological changes of the tumor cell, indicating apoptosis, after administration of 5 μM DAPT, 1 μM RY10-4, or combination of the two for 24 h were identified using probes that stain for apoptosis; such changes were detected by fluorescence microscopy. (**D**) The mean red fluorescence intensity of MDA-MB-231, MCF-7 and SKBR3 cells after treatment were collected. Data are expressed as mean ± SD, *P < 0.05, **P < 0.01, ***P < 0.001.

To determine the role of Notch signaling in mediating drug resistance to RY10-4 in HER2-overexpressing cells, we studied the effect of combining RY10-4 treatment with DAPT, a highly specific γ-secretase inhibitor that blocks Notch endoprotrolysis [20]), on cell viability using two HER2-positive cell lines (SKBR3 and BT-474) and two HER2-negative cell lines (MDA-MB-231 and MCF-7). The anti-proliferation effect was evaluated using the colony formation assay or the MTT assay. RY10-4 at a concentration of 1 μM markedly suppresses cell growth when compared with vehicle control (Figure 4A, Figure S4, Figure S5A–S5B). Greater suppression is observed with the combinational use of DAPT (5 μM) and RY10-4 (1 μM) in both SKBR3 and BT-474 cell lines. In contrast, we did not observe a synergistic effect in the RY10-4-sensitive cell lines, MDA-MB-231 and MCF-7, suggesting that the increased anti-proliferation effects of DAPT and RY10-4 in HER2-overexpressing cells are specifically mediated by inhibiting the Notch pathway.

As expected, untreated cells (vehicle control) exhibit normal shape with clear outlines. Growth of the RY10-4-treated cells is inhibited when compared with control cells and DAPT-treated cells. Further, the cells treated with RY10-4 became rounded and detached. Cell shrinkage and membrane blebbing were also observed (Figure 4B, Figure S5C). These data indicate that cells undergo RY10-4-mediated apoptosis. Notably, RY10-4 markedly induces apoptosis when delivered in combination with DAPT, as measured by Annexin V-PE staining (Figure 4C–4D, Figure S5D). In sharp contrast, DAPT fails to enhance RY10-4-mediated apoptosis in MDA-MB-231 and MCF-7 cells.

**Figure 5:**
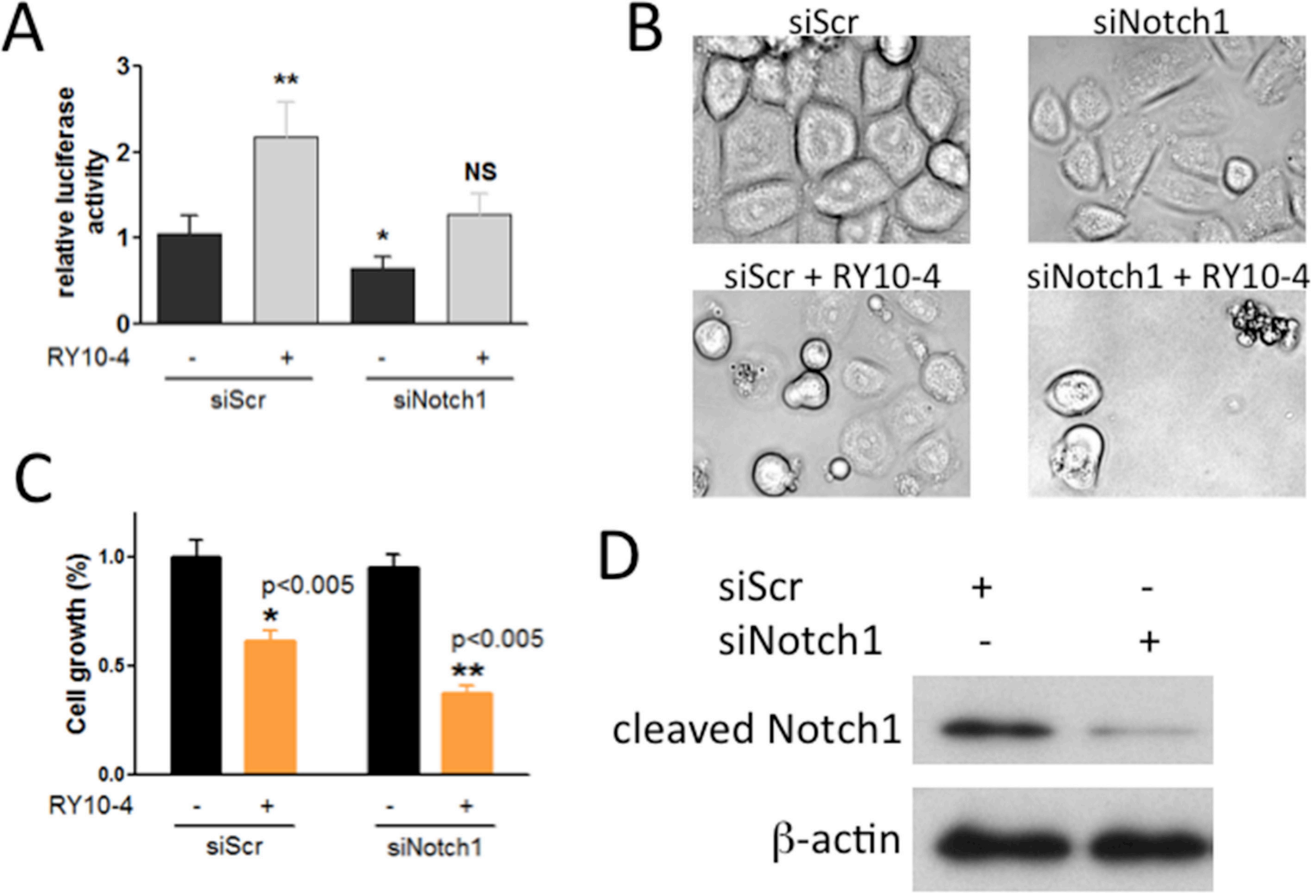
Notch-1 siRNA prevents RY10-4-induced activation of the Notch signaling pathway and causes RY10-4-induced cell death in SKBR3 cells (**A**) SKBR3 cells were co-transfected with the CSL luciferase reporter and scrambled control siRNA or Notch-1 siRNA for 24 h and then treated with RY10-4 or vehicle control. (**B**) Representative micrographs of the cells with 100 nM Notch-1 siRNA treatment, 1 μM RY10-4 treatment, and combinational treatment for 24 h. (**C**) Methylene blue was used to evaluate the cell viability following different treatments. (**D**) Western blot analysis detected Notch-1 and β-actin proteins in SKBR3 cells previously transfected with scrambled siRNA or Notch-1 siRNA for 24 h. Data are expressed as mean ± SD, *P < 0.05, **P < 0.01, ***P < 0.001.

Since DAPT preferentially affects Notch1 activity, we sought to determine whether the RY10-4-induced increase in Notch activation is due to specifically Notch1. To do this, we determined the effect of Notch1-specific knockdown on cell proliferation and apoptosis using si-Notch1. The results show that Notch1 siRNA almost completely abolishes Notch1 protein expression (Figure 5D) and also prevents the increase in CSL reporter activity initially observed upon treating cells with RY10-4 (Figure 5A). In cell proliferation experiments, cell growth is reduced by 40% in response to RY10-4 alone and by more than 60% upon combining RY10-4 with si-Notch1 (Figure 5C). In addition, combination treatment further induces cell apoptosis in SKBR3 cells according to morphological study of cell shape (Figure 5B). These findings show that the addition of a Notch inhibitor to RY10-4 treatment is significantly more effective than RY10-4 alone in suppressing both HER2-overexpressing cell lines.

### Combination of RY10-4 and DAPT inhibit Notch signaling and AKT activation

**Figure 6:**
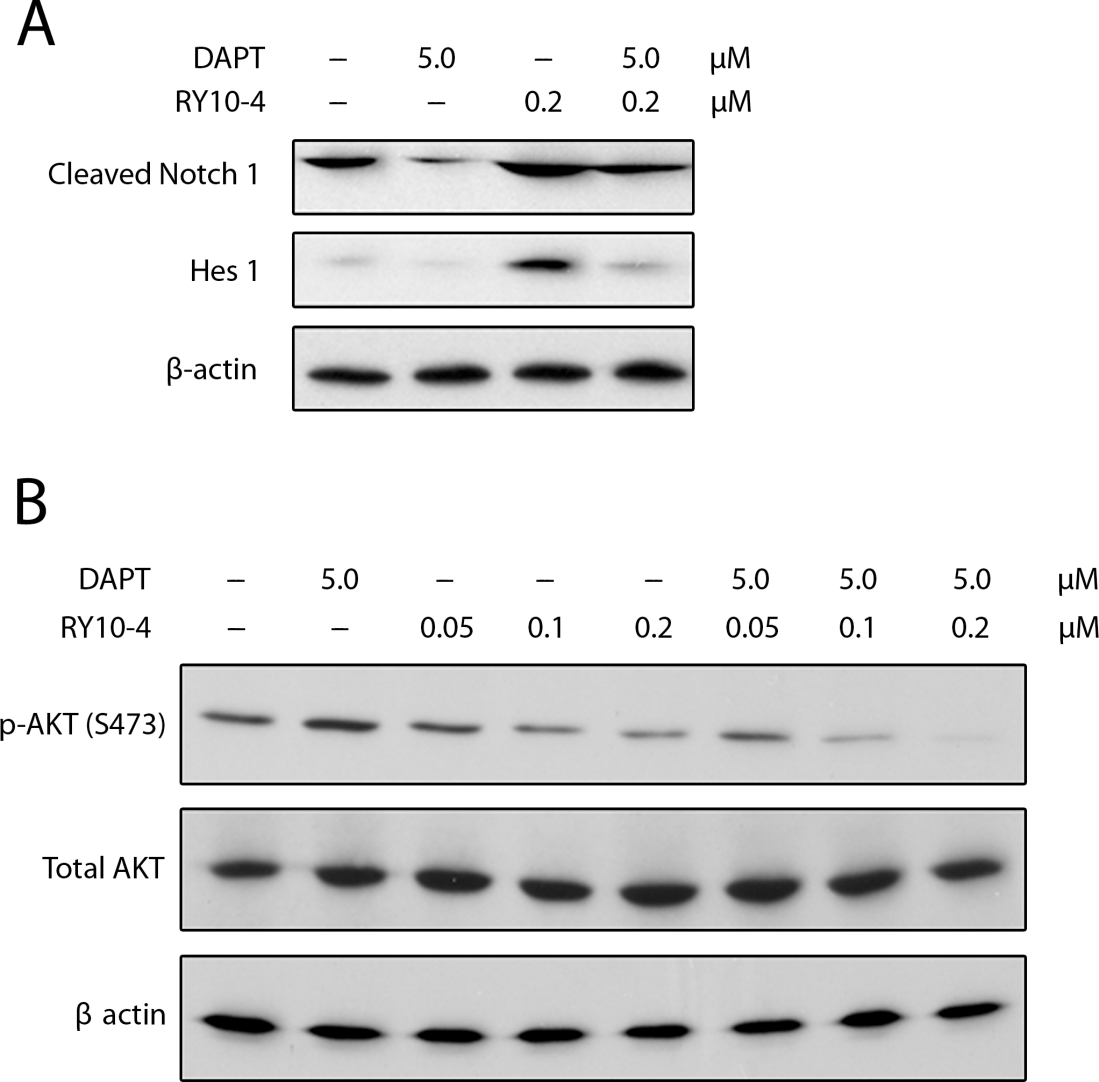
(**A**) DAPT reduces RY10-4-induced expression of NICD and Hes1 in SKBR3 cells. SKBR3 cells were treated with only RY10-4 (0.2 μM), only DAPT (5 μM), or both for 24 h. Protein expression of NICD1 and Hes1 was analyzed by western blot. (**B**) Effects of DAPT and/or RY10-4 on the expression of AKT and p-AKT in SKBR3 cells. SKBR3 cells were treated with only RY10-4 (0.05, 0.1, and 0.2 μM), only DAPT (5 μM), or both for 24 h. The expression of AKT and p-AKT (S473) at the protein level was measured by western blot. Cells treated with vehicle served as control.

Since Notch activates the PI3K/AKT pathway [21, 22] and since AKT activation is necessary for Notch-conferred resistance to apoptosis [23], we assessed the effect of combining RY10-4 treatment with DAPT on Notch signaling and AKT phosphorylation in SKBR3 cells. First, SKBR3 cells express two-fold higher levels of active Notch1 (cleaved Notch1) and eight-fold higher levels of Hes1 protein in response to RY10-4 treatment compared to vehicle control. The increase in cleaved-Notch1 and Hes1 expression is prevented by treatment with DAPT (Figure 6A). We next showed that RY10-4 alone, at a concentration of 0.2 μM, was sufficient to inhibit AKT phosphorylation on Ser473 by 50% in SKBR3 cells, and this increased to 95% with DAPT (Figure 6B). Similar results are seen in BT474 cells (Figure S6). Interestingly, treating either HER2-positive cells with DAPT alone proves ineffective in inhibiting AKT phosphorylation (Figure 6B lane 2 and Figure S6 lane 5), which suggests that the Notch signaling is suppressed in the HER2-amplified cells [14]. Thus, DAPT cannot inhibit AKT phosphorylation through the Notch pathway. Taken together, these data show that the combination of RY10-4 and DAPT inhibits Notch signaling and Notch-stimulated AKT activation.

### DAPT and RY10-4 combination inhibits breast tumor growth *in vivo*

**Figure 7:**
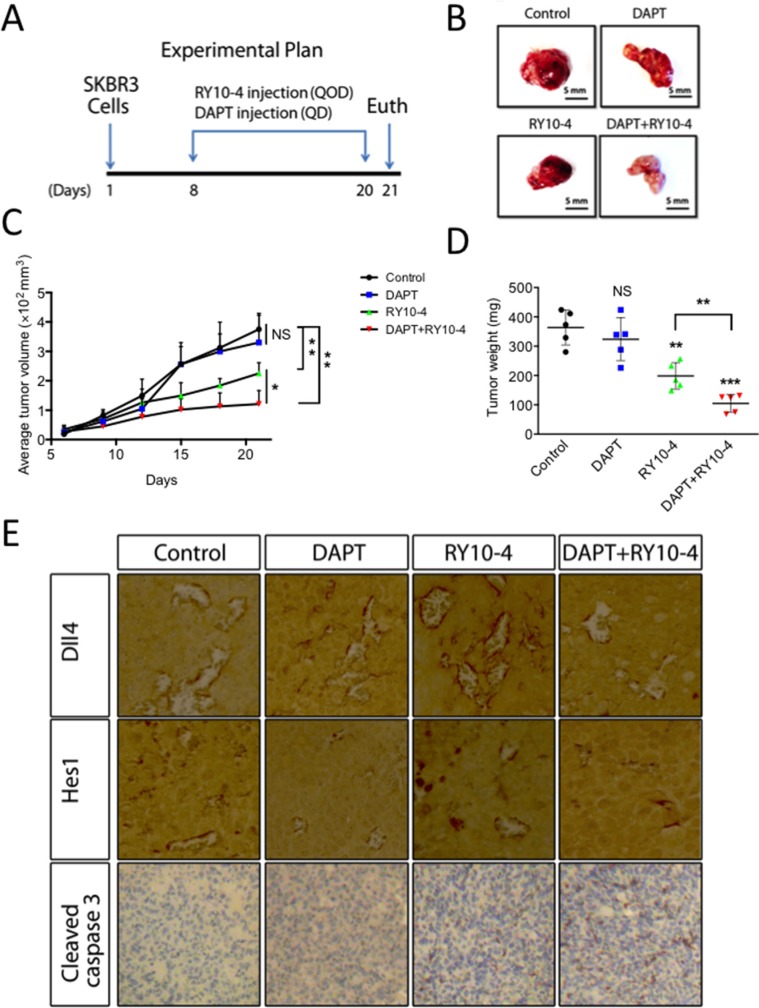
DAPT and RY10-4 inhibit the growth of SKBR3 xenografts BALB/c nude mice were challenged subcutaneously with 1 × 10^6^ SKBR3 cells for seven days, followed by administration with vehicle, DAPT (100 mg/kg, QD), RY10-4 (200 mg/kg, QOD), or DAPT + RY10-4 (*n* = 5 mice per group) for another twelve days. The mice were euthanized, and the tumor tissue was dissected and weighed. (**A**) A schematic diagram depicting the experimental protocol used for tumor xenografts. (**B**) Representative pictures of isolated tumors. (**C**) Measurement of tumor volume. (**D**) Measurement of tumor mass from different groups of mice. Data are expressed as mean ± SD, *P < 0.05, **P < 0.01, ***P < 0.001, versus vehicle control group. (**E**) Tumors were cut into sections, and immunohistochemistry staining was performed. Representative images showing staining for Dll4, Hes1, and cleaved caspase-3 from the control, DAPT-treated, RY10-4-treated, and combination-treated groups are shown at ×400 magnification.

From *in vivo* studies of the effects of treatment with DAPT, RY10-4, combination of DAPT and RY10-4, or vehicle (Figure 7A), we found no statistical difference (*P* = 0.81 and 0.15, respectively) in tumor volume and tumor weight between the DAPT group and the vehicle control group. In contrast, treatment with RY10-4 (mean tumor size 225 mm^3^, mean tumor weight 198 mg) and treatment with a combination of DAPT and RY10-4 (mean tumor size 121 mm^3^, mean tumor weight 105 mg) significantly inhibit SKBR3 tumor progression compared to vehicle-treated animals (mean tumor size 375.3 mm^3^, mean tumor weight 363.4 mg Figure 7C, 7D). Interestingly, we found that, in the combination group, the tumors are whiter than the other groups (Figure 7B), hinting that treatment with DAPT in combination with RY10-4 disturbs angiogenesis in SKBR3 tumors. There was no significant difference in mouse weight between treatment groups in either experiment, suggesting that the combination of DAPT and RY10-4 does not significantly increase systemic toxicity (data not shown). In summary, DAPT treatment enhances the anti-tumor effects of RY10-4, but it exerts no effect if used alone in nude mice bearing SKBR3 tumors.

To evaluate the effects of combined and single-agent treatments on SKBR3 cells, we examined levels of Notch and caspase-3 activity by immunohistochemistry (Figure 7E). Protein levels of HES1 in SKBR3 tumors are decreased in mice treated with DAPT. In contrast, treatment with RY10-4 results in a great increase in both Dll4 and Hes1 expression, suggesting that Notch signaling is activated in SKBR3 xenografts exposed to RY10-4 alone. Interestingly, we found that the combination of DAPT and RY10-4 in treatment helps reverse the induction effects caused by treatment with RY10-4. Basal levels of cleaved caspase-3 are low in SKBR3 tumor tissues and remain low in DAPT-treated tumor tissues. However, we saw a trend in increased induction by RY10-4 treatment, and RY10-4 as a single agent was less effective in inducing cleaved caspase-3 expression than the combination of DAPT and RY10-4. These results suggest that aberrant Notch activation by RY10-4 treatment impairs the anti-tumor effects of RY10-4. Further, combining a Notch inhibitor with RY10-4 in treatment could help to improve the anti-cancer effects of RY10-4.

## DISCUSSION

There are four subtypes of breast cancer based on the expression of ER, progesterone receptor (PR), or HER2: ER, PR+, HER2+; ER, PR+, HER2–; ER, PR–, HER2+; and ER, PR–, HER2– [24]. Among four breast cancer cell lines we tested, the triple negative cell line MDA-MB-231 and the ER+, PR+, HER2– cell line MCF-7 are very susceptible to RY10-4 as measured by MTT and methylene blue methods. To our surprise, The ER^−^, PR–, HER2+ cell line SKBR3 and ER+, PR+, HER2+ cell line BT474 are less sensitive. HER2 positive breast cancer occurs in 25% of breast cancer patients, and poor prognosis is due to activation of the PI3K/AKT survival pathway [25]. Although two HER2-targeted drugs (trastuzumab and lapatinib) have been approved for clinical use by the FDA, most patients still relapse after initially responding to treatment [26–28]. Activation of Notch signaling is a common result of using HER2 inhibitors or a dual EGFR/HER2 inhibitor, such as a tyrosine kinase inhibitor [14, 15]. The Notch pathway, an essential upstream regulator of HER2 signaling, is positively correlated with drug resistance and has a tumor-promoting function in breast cancer [29]. Activation and subsequent nuclear localization of the Notch intracellular domain induces the transcription of the target HER2 gene, which, in turn, activates the PI3K/AKT pathway [30]. Furthermore, other studies have confirmed that AKT activation is necessary for Notch-conferred resistance to apoptosis [23].

Since RY10-4 inhibits the HER2/PI3K/AKT axis, we investigated whether Notch signaling contributes to RY10-4-induced drug resistance in HER2-positive cells. Our results demonstrate that treatment with RY10-4 increases expression of Notch ligands, activating Notch-1^ICD^ and targets by increasing both mRNA and protein levels. The increased activity is abrogated by DAPT, a Notch inhibitor. Importantly, cell proliferation is inhibited more effectively by RY10-4 plus DAPT or Notch1 siRNA than by either agent alone. RY10-4 at a concentration of 0.5 μM alone was sufficient to decrease cell growth by more than 50% in MDA-MB-231 and MCF-7 cells, but only around 30% in HER2-amplified SKBR3 and BT47 cells. This growth inhibition rises to 50% upon combination with DAPT or Notch1 siRNA. However, we did not observe a synergistic effect between DAPT and RY10-4 in MDA-MB231 and MCF-7 cell lines. This suggests that the increased anti-proliferative effects of DAPT when combined with RY10-4 are specifically mediated by reversal of RY10-4-indued drug resistance. In addition, we show that RY10-4 alone is sufficient to inhibit AKT phosphorylation on Ser473 by 50%, which dramatically increases to 95% with DAPT. Our *in vivo* data also demonstrates the synergistic effect of combinationtreatment, which decreases tumor growth 20–30% more than compared to RY10-4 alone. Furthermore, we found the decrease in cell proliferation is due to a significant increase in apoptosis as RY10-4 plus DAPT increases annexin V staining in SKBR3 cells and induces cleaved-caspase3 expression in tumor tissue.

The mechanisms responsible for Notch-related drug resistance are complex and still poorly understood. Some studies have demonstrated that Notch signaling is involved in the epithelial-mesenchymal transition in drug resistant cancer cells [31]. Other studies showed that abnormal Notch signaling might contribute to carcinogenesis by regulating the formation of cancer stem cells [32]. However, the mechanisms by which Notch signaling regulates the sensitivity of breast cancer cells to RY10-4 are still under study.

Our results reveal that RY10-4 is a promising drug candidate for the treatment of different types of breast cancer. Cell proliferation of triple-negative and ER-positive breast cancer is significantly inhibited upon treatment with RY10-4. For HER2-positive cell lines, we found that increased activation of Notch signaling upon RY10-4 treatment can cause drug resistance. Combination treatment with a Notch inhibitor increases the efficacy of RY10-4 and prevents resistance. This work helps us gain important insight into the molecular pathways involved in the sensitivity of breast cancer cells to RY10-4, and it allows us to rationally design successful combination therapies for breast cancer treatment.

## MATERIALS AND METHODS

### Reagents

RY10-4 (Figure S1) was synthesized previously in our laboratory [6], and the structure was confirmed by NMR and MASS. Purity (95%) was measured by HPLC analysis. RY10-4 was dissolved in dimethyl sulfoxide (DMSO) to make a 10 μM stock solution, and this was stored at −20°C. The working dosage was freshly prepared in basal medium with a final DMSO concentration of less than 0.1%. The antibody against β-actin was purchased from Santa Cruz Biotechnology (Santa Cruz, CA, USA), and the antibodies against AKT, p-AKT, Notch1, NICD, and HES1 were purchased from Cell Signaling Technology (Beverly, MA, USA). The antibody against Dll4 was purchased from Abcam (Cambridge, MA, USA). Horseradish peroxidase (HRP)-conjugated anti-mouse IgG and anti-rabbit IgG were purchased from Santa Cruz Biotechnology (Santa Cruz, CA, USA). All other chemicals were obtained from Sigma Aldrich (St. Louis, MO, USA).

### Cell culture

MDA-MB-231 and MCF-7 human breast cancer cells were obtained from American Type Culture Collection. SKBR3 and BT474 human breast cancer cells were kindly provided by Dr. Qin Yan (Department of Pathology, Yale School of Medicine). They were propagated in RPMI-1640 medium supplemented with 10% (v/v) fetal bovine serum (FBS), 100 U/ml penicillin, and 100 μg/ml streptomycin. All cell lines were maintained at 37°C in a humidified atmosphere of 5% CO_2_.

### Methylene blue cell proliferation assay

Exponentially growing cells (1 × 10^4^) were plated in a 48-well plate and treated with various concentrations of RY10-4 dissolved in DMSO (giving a final DMSO concentration of ≤ 0.1%) in media after 24 h growth. Incubation was carried out at 37°C for 24 h. Controls received vehicle in DMSO at a concentration equal to that of RY10-4 treated cells. Media was removed, and cells were washed with phosphate buffered saline (PBS), air dried, stained with 0.3 ml methylene blue (2.5 g in 250 ml EtOH + 250 ml H_2_O), left at room temperature for 2 h, and then washed with water. To each well, 0.5 ml 1% Sarkosyl was added, and plates were rotated at room temperature for 3 h. Transferring 150 μl to a 96-well plate allowed for determining the OD using a plate reader at 595 nm. Cell viability assays were performed with three independent experiments.

### MTT cell proliferation assay

The effect of RY10-4 on breast cancer cell proliferation was also assessed by using the 3-(4,5-dimethylthiazol-2-yl)-2,5-diphenyl-2H-tetrazolium (MTT)bromide assay. Exponentially growing cells (0.5 × 10^4^) were plated in 96-well plates and treated with concentrations of RY10-4 in media (giving a final DMSO concentration of ≤ 0.1%) after 24 h of growth. Incubation was carried out at 37°C for 24 h. Controls received DMSO vehicle at a concentration equal to that of drug-treated cells. MTT solution was added to each well (2.5 mg/ml) and incubated for 4 h. Supernatants were removed from the wells, and the reduced MTT bromide dye was solubilized in 200 μl/well DMSO. Absorbance at 570 nm was determined on a plate reader.

### Colony-formation assay

Breast cancer cell lines were plated on 6-well plates at a density of 1 × 10^3^ cells per well and treated with RY10-4 and/or DAPT. Media was changed after 24 h of incubation, and colonies were observed over seven days. Colonies were then stained with 0.5% of methylene blue in 50% ethanol for 2 h. Quantification of colony formation was processed using the “ColonyArea” plugin on ImageJ [34].

### RNA isolation and qPCR

Total RNA was extracted from cells using the RNeasy Mini kit (Qiagen, CA, USA) according to the manufacturer's protocol. Total cDNA was reverse-transcribed from the total RNA with random hexamers using the MultiScribe^™^ Reverse Transcriptase Kit (Applied Biosystems, CA, USA) according to the manufacturer's recommendations. Analysis of transcript copy number relative to that of glyceraldehyde 3-phosphate dehydrogenase (GAPDH), an endogenous control, was carried out by quantitative real-time PCR (BIO-RAD iQ^™^5, USA) using iTaq^™^ SYBR Green. Gene expression was determined by normalizing to reference genes using the comparative C_T_ method. A list of the oligonucleotide sequences used for qPCR can be found in Table 1.

**Table 1:** Primers used for qPCR

Name	Sequence
h Hey1	F: ACCCCAAACTCCGATAGTCC
	R: TGAGCTGAGAAGGCTGGTAC
h Hes1	F: ATTCCTCGTCCCCGGTGGCT
	R: CAGCTTGGAATGCCGCGAGCT
h Notch1	F: CAGCGAATCCGAGGACTATG
	R: CAGGCGTGTTTGTTCTCACAG
h Notch4	F: CACGTGAACCCATGTGAGGTC
	R: CACAGTGGAATCCTCCAGGT
h Jagged1	F: TCGCTGTATCTGTCCACCTG
	R: AGTCACTGGCACGGTTGTAG
h Dll4	F: TGCAGGAGTTCATCAACGAG
	R: GAAATTGAAGGGCAGTTGGA

### Luciferase reporter assays

The CSL (CBF1/RBP-J) luciferase reporter, (a gift from Dr. Yungchi Cheng, Department of Pharmacology, Yale School of Medicine), which contains a firefly luciferase gene under the control of the multimerized CSL responsive element upstream of the minimal promoter, was transiently transfected into cells using the FuGene (Roche, San Francisco, USA) transfection reagent. The pRL plasmid, a vector that constitutively expresses the Renilla luciferase gene under the control of the cytomegalovirus promoter, was used as an internal control. Total DNA was kept constant by adding empty vector as needed. After transfection, cells were treated with RY10-4, DAPT, or si-Notch1 for 6 hours. All transfections were carried out in triplicate. The luciferase activity was measured with the luciferase assay kit (Promrga, USA) and the Tecan FARCyte luminometer (GE Healthcare, USA) according to the manufacturers' instructions.

### *In vitro* transfection of siRNA

Cells were transfected with siRNA using Oligofectamine (Life Technologies, USA), performed according to the supplier's instructions. One day prior to transfection, 2 × 10^5^ cells per six-well plate were seeded without antibiotics, corresponding to a density of 40%–50% at the time of transfection. Cells were treated with siNotch1 or control, a scrambled sequence, at a concentration of 100 nM. After each treatment, cells were incubated at 37°C for 4 h followed by addition of fresh culture media. Cells were harvested 24 h after transfection for protein analysis.

### Western blot analysis

Cells were harvested and lysed on ice for 30 min in lysis buffer containing 50 mM Tris-Hcl, pH 8.0, 150 mM NaCl, 20 mM EDTA, 50 mM NaF, 1% NP-40, and 0.02% NaN_3_ with protease inhibitor (1 mM phenyl-methanesulfonyl fluoride (PMSF) and, 1 μg/ml aprotinin) to prevent proteolysis and dephosphorylation. After centrifugation at 16099 g for 10 min, the supernatant was harvested as the total cellular protein extract. Protein concentration was determined using the Pierce BCA protein assay kit (Thermo Scientific, USA). The total cellular protein extracts were separated by SDS-PAGE and transferred to polyvinyldifluoride (PVDF) membranes (Millipore, USA). The membranes were blocked with 5% (w/v) nonfat dry milk in TBST (1 M Tris buffer saline, pH 7.4, 5 M NaCl, and 0.1% Tween-20) for 1 h at room temperature and incubated overnight at 4°C with primary antibody. Blots were washed three times in TBST buffer, followed by incubation for 1 h at room temperature with the corresponding HRP-linked secondary antibodies. Specific proteins were visualized using enhanced chemiluminescence reagent (Thermo Scientific, USA).

### Annexin V labeling

Cells were incubated with RY10-4 and/or DAPT for 24 h. After treatment, cells were washed twice with PBS and once with binding buffer (Abcam, USA). After adding 5 μl of Annexin V-PE to the media, cells were incubated at room temperature for 15 min in the dark. Analysis was performed using a fluorescence microscope (Olympus IX71) with the filter set for rhodamine.

### *In vivo* tumor xenograft study

Six-week-old female nude mice (BALB/c) were inoculated subcutaneously at the right flank with 2 × 10^6^ SKBR3 cells suspended in 0.2 ml PBS (pH 7.4). When tumors reached an average diameter of 3 mm (usually in seven days), the mice were randomized into 4 groups and administered DAPT, RY10-4, combination of DAPT and RY10-4, or vehicle. Each group consisted of five animals. RY10-4 was administered intraperitoneally (i.p.) every other day at 200 mg/kg, and DAPT was administered i.p. every day at 100 mg/kg. The control group was given vehicle (Figure 6A). Tumor size was measured using calipers, and tumor volumes (mm^3^) were calculated according to a standard formula: width^2^ × length/2. Upon termination of the experiment, the mice were sacrificed, and the tumors were excised for weighing.

### Immunohistochemistry

After treatment over 14 days, the mice were sacrificed by euthanasia. The tissues were fixed overnight in 4% paraformaldehyde, dehydrated, and coated with wax. Tissue sections were sliced to 4 μm in thickness and dyed with the primary antibody. Results were captured by the Nikon TS100 microscope.

### Statistics

The data represent mean ± SD from independent experiments. Statistical analysis was performed using the student's *t* test and the ANOVA post test. The level of significance was set at *P* < 0.05.

## SUPPLEMENTARY MATERIALS FIGURES


