# Supplementary material for: Combination therapy of RY10-4 with the γ-secretase inhibitor DAPT shows promise in treating HER2-amplified breast cancer
Source: Oncotarget. 2015 Dec 26;7(4):4142–54. doi: 10.18632/oncotarget.6769 (PMC4826195; doi:10.18632/oncotarget.6769)
Supplement: Supplementary file 1 [file oncotarget-07-4142-s001.pdf]

Combination therapy of RY10-4 with the γ-secretase inhibitor DAPT shows promise in treating HER2-amplified breast cancer

Supplementary Materials

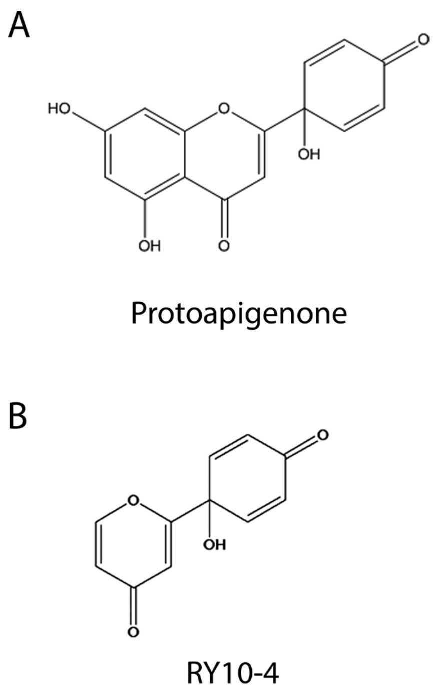

Supplementary Figure S1: Chemical structures of (**A**) Protoapigenone and (**B**) RY10-4.

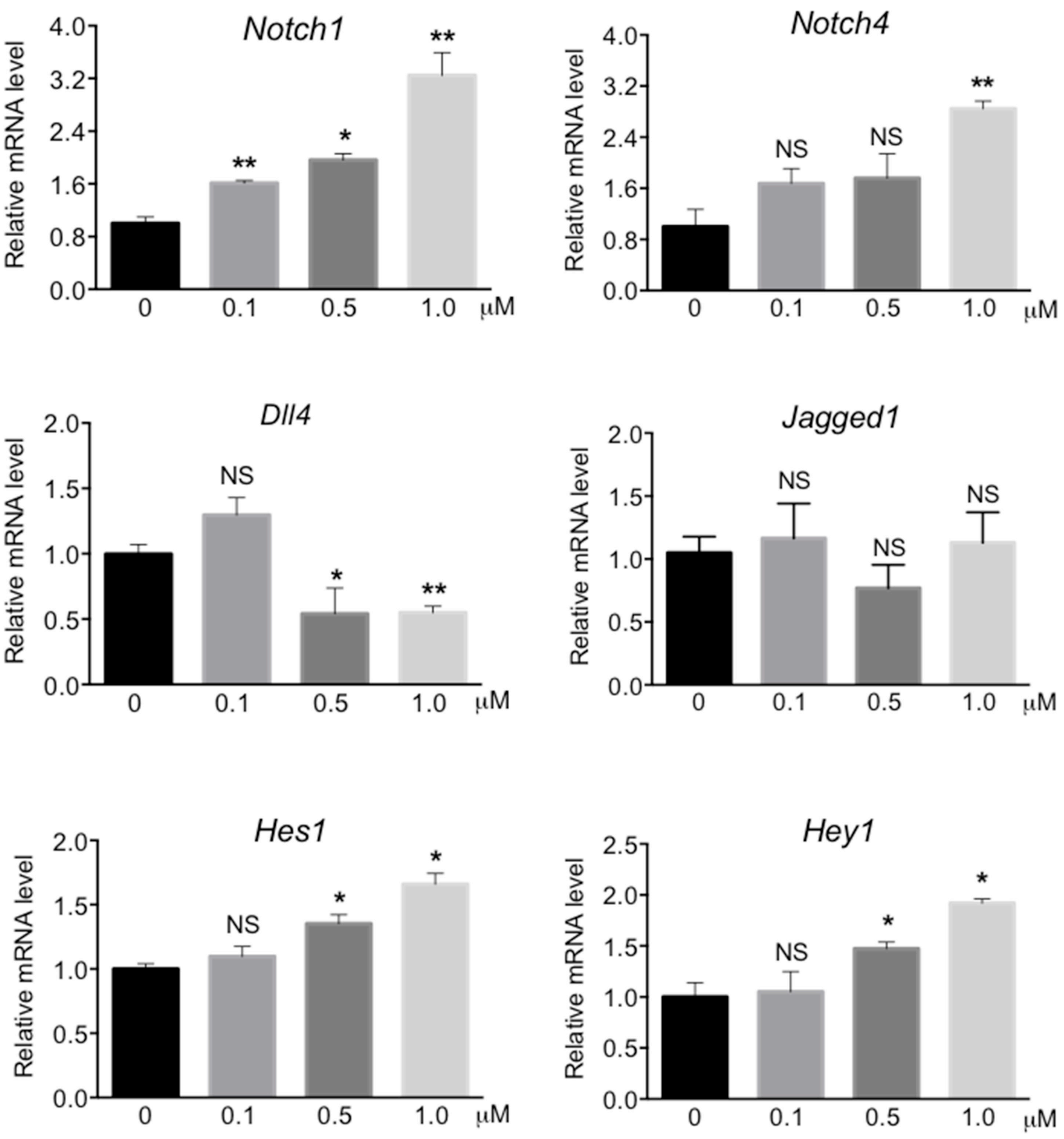

Supplementary Figure S2: RY10-4 induces Notch signaling in BT474 cells. Shown is qPCR analysis of Notch1, Notch4, Dll4, Jagged1, Hes1, and Hey1 gene expression in BT474 cells after treatment with different concentrations of RY10-4 over a 6-h time course. Expression of Notch signaling genes was normalized to that of GAPDH. Data represent mean ± SD, $*P < 0.05$, $**P < 0.01$, $***P < 0.001$, versus vehicle control.

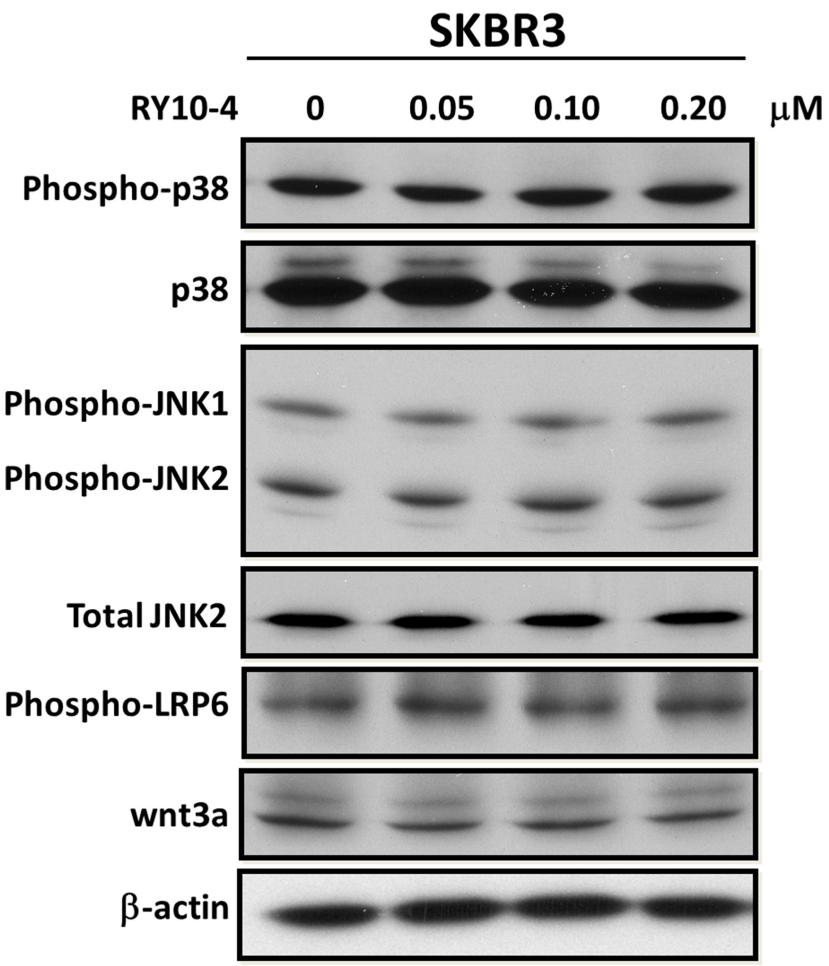

Supplementary Figure S3: RY10-4 has no effect on MAPK or Wnt signaling. SKBR3 cells were treated with 0.05, 0.1, or 0.2 µM RY10-4 for 24 h. Western blotting was performed to detect MAPK pathway-related p-p38, total p38, p-JNK1/2, and total JNK1/2, as well as Wnt pathway-related p-LRP6, Wnt 3a, and β-actin.

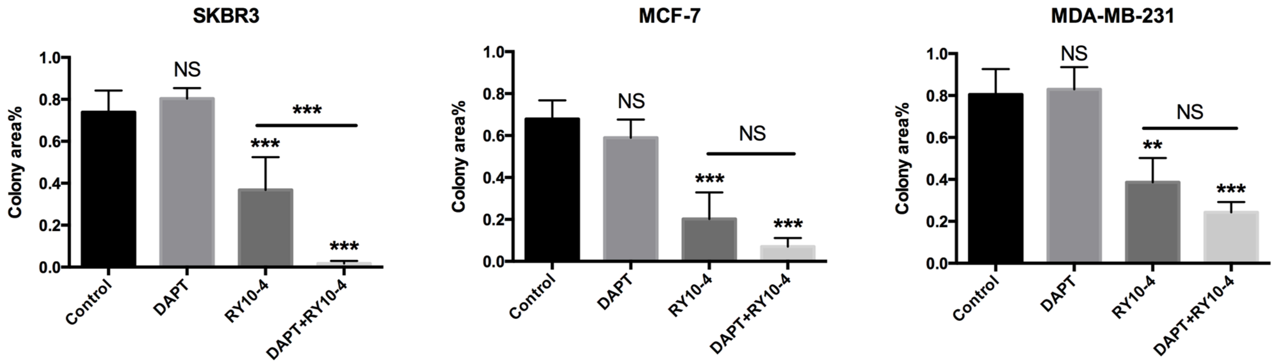

Supplementary Figure S4: Quantification of data from the colony formation assay of Figure 4A.

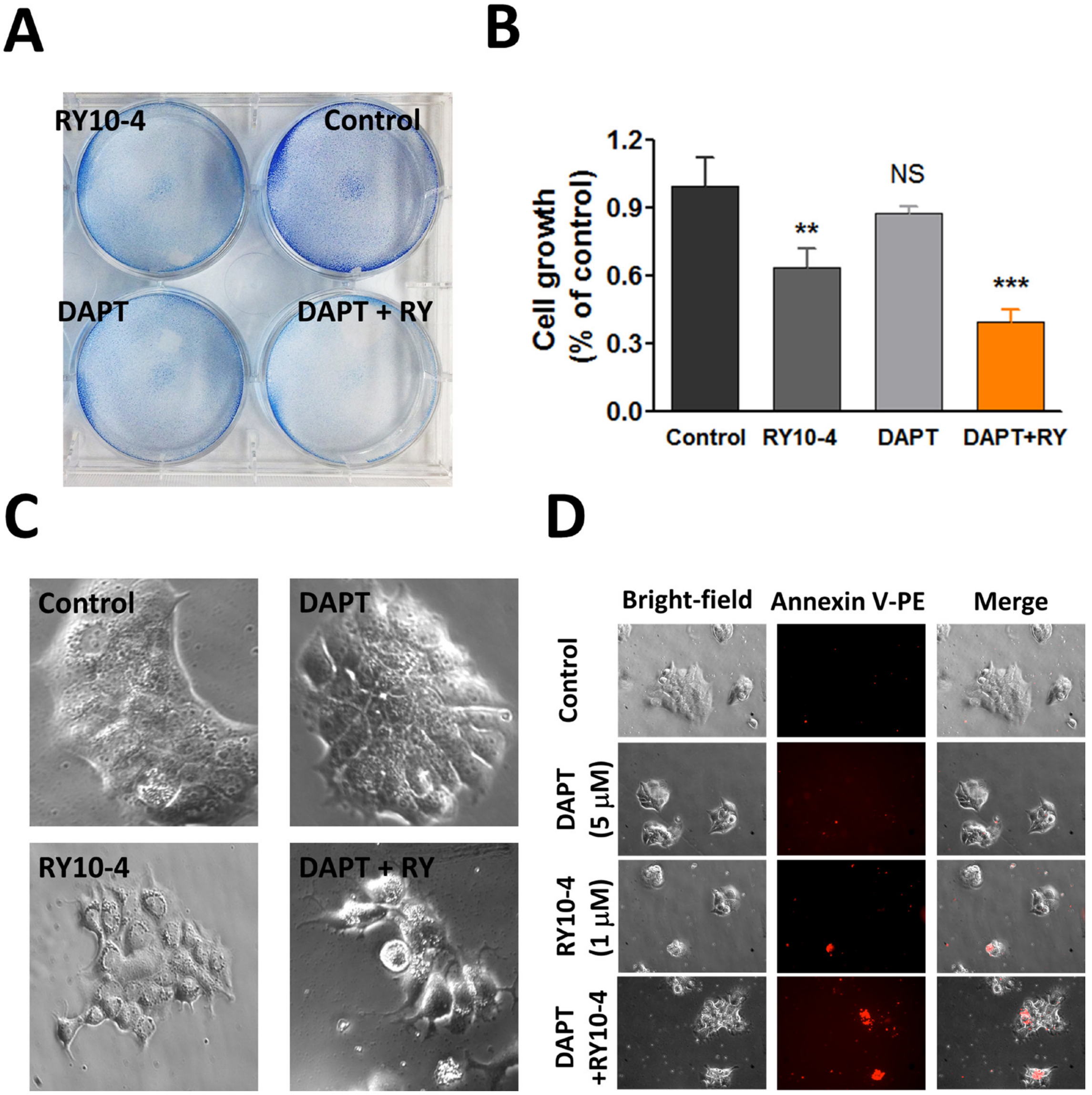

Supplementary Figure S5: DAPT and RY10-4 have additive effects on the proliferation of BT474 cells. The effect of DAPT (5 μM), RY10-4 (1 μM), and combination of the two on the growth of BT474 cell was investigated by (**A**) the colony formation assay and (**B**) the MTT assay. (**C**) Shown are representative micrographs of BT474 cells with 5 μM DAPT treatment, 1 μM RY10-4 treatment, or both for 24 h. (**D**) Annexin-PE staining was used to evaluate cell viability following treatment of different samples. Morphological changes indicative of apoptosis, caused by treatment with 5 μM DAPT, 1 μM RY10-4, or combination of the two for 24 h, were compared by staining cells with an apoptosis probe and detected by fluorescence microscopy. Data repesent mean ± SD, *$P < 0.05$, **$P < 0.01$, ***$P < 0.001$.

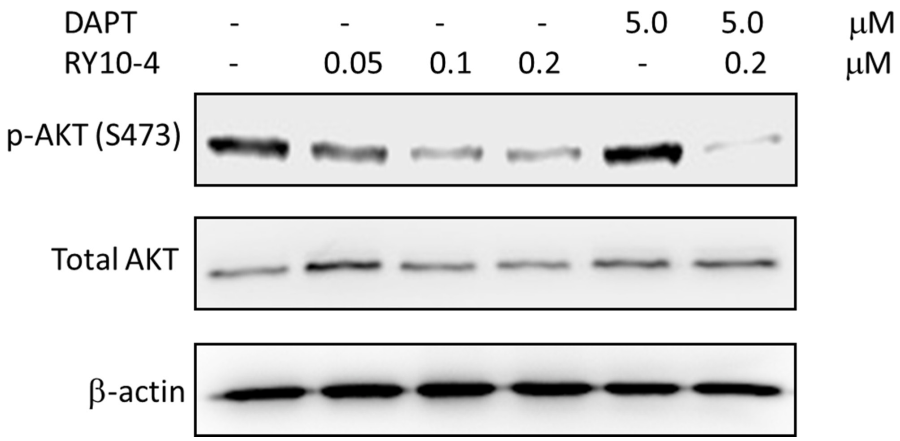

Supplementary Figure S6: DAPT and RY10-4 have additive effects on suppressing the expression of AKT and p-AKT in BT474 cells. BT474 cells were treated with only RY10-4 (0.05, 0.1, and 0.2 μM), only DAPT (5 μM), or both for 24 h. Protein expression of AKT and p-AKT (S473) was measured by western blotting. Cells treated with vehicle served as control.
